# In Vitro Cytotoxicity of Trastuzumab (Tz) and Se-Trastuzumab (Se-Tz) against the Her/2 Breast Cancer Cell Lines JIMT-1 and BT-474

**DOI:** 10.3390/ijms22094655

**Published:** 2021-04-28

**Authors:** Priyanka Bapat, Debalina Goswami Sewell, Mallory Boylan, Arun K. Sharma, Julian E. Spallholz

**Affiliations:** 1 Nutritional Sciences, College of Human Sciences, Texas Tech University, Lubbock, TX 79409, USA; bapat.priyanka@gmail.com (P.B.); goswami.rima1985@gmail.com (D.G.S.); malloryboylan1@gmail.com (M.B.); 2 Department of Pharmacology, Penn State Cancer Institute, CH72, Penn State College of Medicine, 500 University Drive, Hershey, PA 17033, USA; aks14@psu.edu

**Keywords:** human epidermal growth factor receptor 2 (Her/2), epidermal growth factor receptor (EGFR), selenium (Se), antibody drug conjugate (ADC), monoclonal antibody (mab), Herceptin^®^, Trastuzumab (Tz), Kadcyla^®^ (T-DM-1), superoxide (O_2_^•−^), reduced glutathione (GSH)

## Abstract

Her/2+ breast cancer accounts for ~25% mortality in women and overexpression of Her/2 leads to cell growth and tumor progression. Trastuzumab (Tz) with Taxane is the preferred treatment for Her/2+ patients. However, Tz responsive patients often develop resistance to Tz treatment. Herein, redox selenides (RSe-) were covalently linked to Tz using a selenium (Se)-modified Bolton–Hunter Reagent forming Seleno-Trastuzumab (Se-Tz; ~25 µgSe/mg). Se-Tz was compared to Tz and sodium selenite to assess the viability of JIMT-1 and BT-474 cells. Comparative cell viability was examined by microscopy and assessed by fluorometric/enzymatic assays. Se-Tz and selenite redox cycle producing superoxide (O_2_^•−^) are more cytotoxic to Tz resistant JIMT-1 and Tz sensitive BT-474 cells than Tz. The results of conjugating redox selenides to Tz suggest a wider application of this technology to other antibodies and targeting molecules.

## 1. Introduction

In 1978, Carpenter et al. discovered Epidermal Growth Factor Receptor 1 (EGFR 1) [1]. Following the discovery of EGFR 1, a group of scientists at the Massachusetts Institute of Technology (MIT) discovered the “neu” gene, known as Human Epidermal Growth Factor Receptor 2 (Her/2). Her/2 encodes a 185 kD transmembrane glycoprotein, a member of the family of EGFR receptors. The EGFR family comprises four homologous receptors—ErbB-1/Her/1/EGFR [2], ErbB-2/Her/2/neu [3], ErbB-3/Her/3 [4], ErbB-4/Her/4 [5]. These receptors have two cysteine rich binding sites, a transmembrane lipophilic segment and an intracellular tyrosine kinase domain [6]. Self-activation of these receptors by either homo- or heterodimerization initiates the complex downstream signaling cascade and cell division. All the receptors except Her/2 bind EGFR via the extracellular domains but it is preferential dimerization that initiates cell division [7]. Normal breast cells express ~20,000 Her/2 receptors/cell, whereas cancer cells have >100 X the expression of the Her/2 receptors/cell. Blocking dimerization and signal transduction was viewed as a therapeutic approach to treat Her/2+ breast cancer. The selected blocking dimerization agent for Her/2 was a monoclonal antibody, Trastuzumab (Tz), generated and further developed by Slamon [8,9] and made commercially and clinically available by scientists at Genentech, South San Francisco, CA [10]. In September 1998, the US FDA approval was granted for Tz, Herceptin^®^ treatment of Her/2+ breast cancer [11]. This Tz drug resistance is clinically prevalent in Her/2+ patients, and Genentech has sought to remedy the resistance by covalently attaching emtansine, a cytoskeletal inhibitor to Tz, forming the ADC, Kadcyla^®^ (T-DM-1). This ADC was clinically approved for treating patients with Tz resistance and metastasis in February, 2013 [12]. 

**Figure 1:**
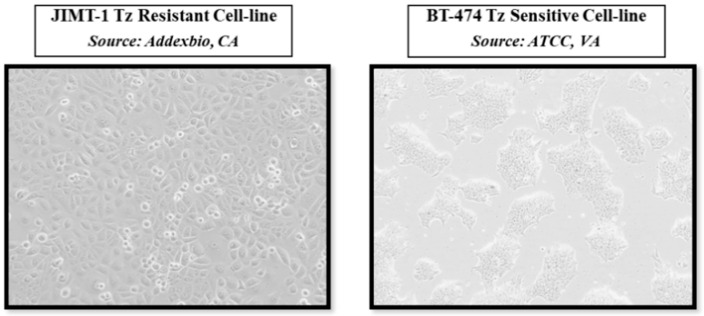
Original morphology of Se-Trastuzumab treated JIMT-1, Trastuzumab resistant and BT-474, Trastuzumab sensitive cell lines. Doubling cell division times for JIMT-1 and BT-474 cells were approximately 30 and 100 h, respectively.

This is the first research that reports the development of a redox selenium (Se) containing ADC of Tz and its therapeutic in vitro comparison to Tz against Herceptin^®^ resistant and Herceptin^®^ sensitive breast cancer cell lines. We covalently attached redox Se to Tz via a modified Se-containing Bolton–Hunter [13] reagent followed by exhaustive dialysis. Unlike Tz, Se-Tz generates superoxide (O_2_^•−^), as measured in vitro or ex vivo, by oxidation of reduced glutathione (GSH) and other thiols and one-electron reduction, as described by Chaudière et al. [14]. Selenite, known for its concentration dependent toxicity to all cells by also generating O_2_^•−^ from GSH oxidation, as described by Seko et al. [15], was congruently compared to the cytotoxicity of Tz and the ADCs against the Tz resistant JIMT-1 and Tz sensitive BT-474 cell lines (Figure 1) over both concentration and time variables assessed by photographic, fluorescent and enzymatic assays of cell viability.

## 2. Results

### 2.1. Selenium Conjugation of Trastuzumab with the Se-Modified Bolton–Hunter Reagent

**Figure 2:**
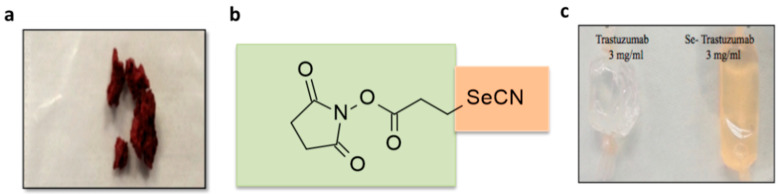
(**a**) Se-BHR: selenium conjugating Bolton–Reagent; (**b**) chemical structure of Se-BHR; (**c**) control and selenium labeled Tz following exhaustive dialysis, pH 7.4.

**Figure 3:**
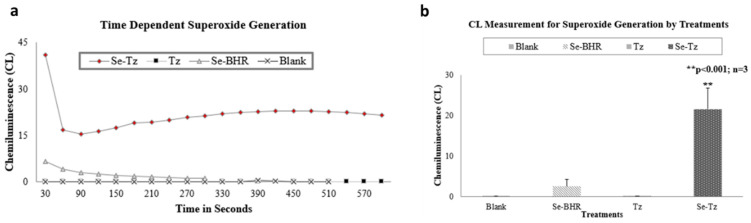
(**a**) O_2_^•−^ CL generated by treatments over time. Figure (**b**) is the sum total of O_2_^•−^ CL generated by each of the treatments over 5 min. For the measurement of superoxide generation, 330 μL of Se-Tz was added to 500 μL of CL cocktail and similar steps were followed for the native Tz (330 μL) and Se-BHR (50 μL) in PBS. Mean CL generation by Se-BHR (20 mg/mL), Tz (protein concentration 3 mg/mL) and Se-Tz (protein concentration 3 mg/mL) was 2.53 ± 1.68, 0.05 ± 0.036 and 21.5 ± 5.17 CLU (** *p* < 0.001), respectively.

The colorless Tz clinical antibody, Herceptin^®^, following redox Se conjugation by the red colored Se-Bolton–Hunter Reagent (Se-BHR) (Figure 2a,b) in pH 8.5 borate buffer produced an orange colored Tz antibody as shown in Figure 2c. The mabs were subjected to exhaustive dialysis in PBS buffer, pH 7.4, and Se conjugation to Tz was then analyzed by inductively coupled plasma mass spectrometry (ICP-MS) by TraceAnalysis, Inc., Lubbock, TX. The Se concentration for dialyzed Se-Tz was calculated to be 25.8 µgSe/mg protein after 72 h of conjugation in borate buffer and the control Tz Se concentration was calculated to be <0.35 µgSe/mg (Figure 3). 

### 2.2. Chemiluminescence (CL) Assay

Generation of O_2_^•−^ by Se-Tz and Tz was measured in vitro by a Lucigenin CL assay. In the presence of GSH, inorganic selenite, but not selenate, also generates superoxide as first reported by Seko et al. and later in 1991, Yan et al. showed the generation of O_2_^•−^ by various organic selenium compounds in the presence of GSH and other thiols [16,17]. In this study, we labeled Tz (Herceptin^®^) with reduceable selenides (RSe^−^) using a Se-BHR and report the redox cycling by Se-Tz but not Tz in the presence of GSH at 37 °C in Figure 3. 

### 2.3. Visual Assessment of the Morphological Changes in JIMT-1 and BT-474 Cell Lines Following Treatments

**Figure 4:**
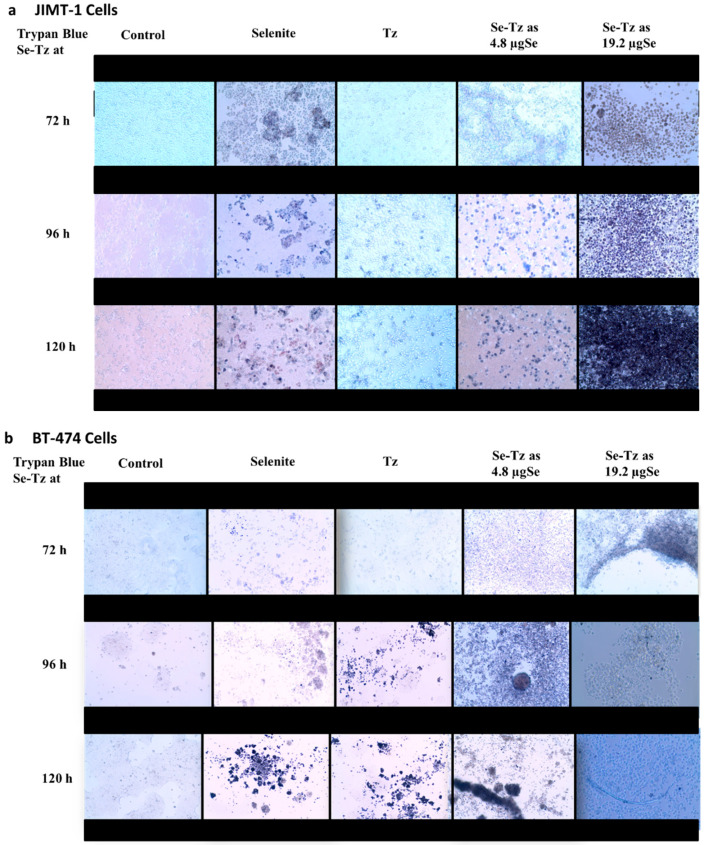
20X Microphotographs of Trypan Blue Stained Cells after Treatments. (**a**) JIMT-1 cells photographed at the concentrations and times indicated; (**b**) BT-474 cells photographed at the concentrations and times indicated. Selenite was used as a redox toxic positive control at a concentration of 10 µgSe/well. Selenite at concentrations less than 10 µgSe/well had no visible effect on cancer cell lines under these experimental conditions.

JIMT-1 and BT-474 cells (Figure 1) were seeded at a density of 1 × 10^5^ cells/well in 24-well plates and were treated with Se-Tz at 4.8 and 19.2 µgSe/well, Tz dose was matched to the equal volume of the highest dose of Se-Tz treatment and with selenite, 10 µgSe/well for 72, 96 and 120 h at which times cells were photographed at 20X magnification after adding Trypan Blue (Figure 4). The photographs in Figure 4a,b visually demonstrate the increasing cytotoxicity of Se-Tz in both cell lines with increasing concentration (left to right) and with increasing time of treatment (top to bottom). Against both cell lines, the classic dose and time dependency of a toxin was observed—in this case, redox active Se-Tz and sodium selenite. Control and Tz cells photographically show only minor if any differences in comparison to Se treated cells.

### 2.4. Superoxide Generation Ex Vivo by DHE: Visual Micrographs and Fluorescence Assay

**Figure 5:**
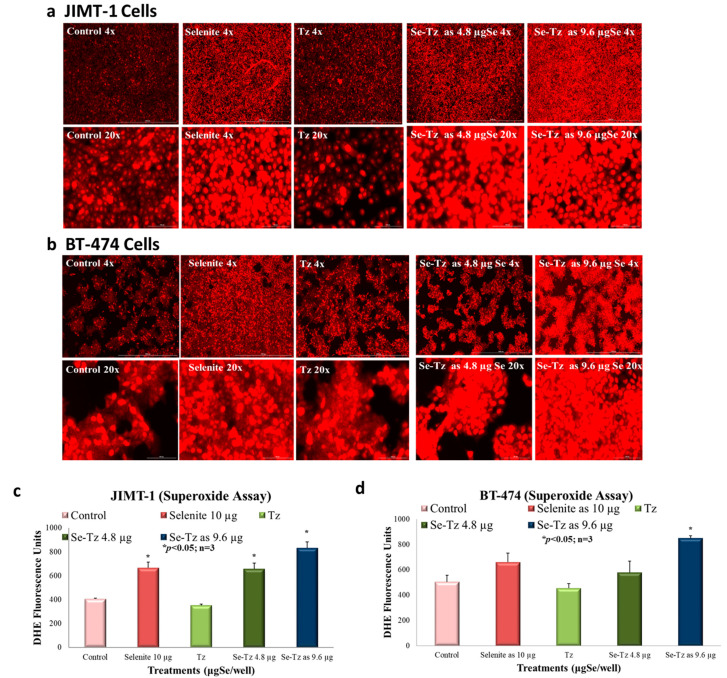
Superoxide Generation by Dihydroethidium (DHE) detection after selenium treatments. (**a**,**b**) Photographs of superoxide staining of JIMT-1 & BT-474 cells after Se treatments at magnifications of 4X= 1000 µm; 20X= 100 µm; (**c**,**d**) Quantitative measurement of superoxide generation of JIMT-1 & BT-474 cells after Se treatments. After acclimatization for 24 h, 50 units/well of SOD and 100 units/well of catalase were added to all wells in media. All cells, control, selenite, Tz and Se-Tz were appropriately treated and then DHE was added to the cells at a final concentration of 10 μM in media. Selenite was used as a redox toxic positive control at a concentration of 10 µgSe/well. Selenite at concentrations less than 10 µgSe/well had no visible effect on cancer cell lines under these experimental conditions. The fluorescence was measured using a microtiter plate reader with an excitation at 520 nm and emission at 610 nm.

JIMT-1 and BT-474 cells were seeded at a density of 2 × 10^5^ cells/well in 24-well plates followed by treatment with Tz and 4.8 and 9.6 µgSe/well Se-Tz, and selenite, 10 µgSe/well. Each well was pretreated with 100 and 50 units of SOD and catalase added to each well, respectively. At 30 min after treatments, 10 µL of DHE, dihydroethidium (Sigma #D7008), was added and cells were photographed. The red color in Figure 5a,b and its intensity are qualitatively and quantitatively indicative of superoxide generation. The fluorescence intensity after photography was measured at an excitation wavelength of 520 nm and 610 nm emission using the microtiter plate reader (BioTek Synergy H1, Winooski, VT, USA). Photographs of the JIMT-1 and BT-474 cells at 4X and 20X magnification demonstrate the red fluorescence of the O_2_^•−^ generated with and without Se treatments. Greater O_2_^•−^ generation was observed at the higher concentrations of Se-Tz, 9.6 µgSe/well, followed by selenite at 10 µgSe/well. After the photographic comparison of Se-Tz induced O_2_^•−^ generation by DHE staining, fluorescence was quantitatively measured using spectrophotometry, revealing the comparative quantitative mean fluorescence intensity observed photographically. The greater DHE fluorescence intensity of the Se-Tz treated JIMT-1 (Figure 5c) and BT-474 (Figure 5d) cells was consistent with the photographic comparisons. Se-Tz at 9.6 µgSe/well demonstrated a significant (*p* < 0.05) increase in fluorescence—850 ± 20 fluorescent units compared to control cells, 504 ± 53 fluorescent units. Native Tz treated cells did not show higher fluorescence intensity as compared to control cells (*p* > 0.05). Selenite treated cells also revealed higher, 661 ± 73, DHE fluorescence units, as compared to control, 504 ± 53, and native Tz, 455 ± 36, fluorescent units, which were significantly different (*p* < 0.05). A complete 96-well blank with all chemicals added except cells and with identical treatments showed no fluorescence, indicating the fluorescence photographed and fluorometrically measured O_2_^•−^ was entirely generated intracellularly. 

### 2.5. H_2_O_2_ Generation Ex Vivo by Dichlorofluorescein (DCFH-DA): Visual Micrographs

**Figure 6:**
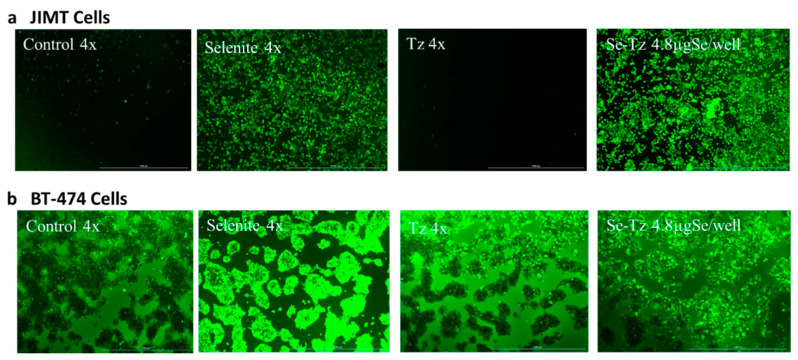
Photographic hydrogen peroxide detection by DCFH-DA after selenium treatments at magnifications of 1000–1010 µm. (**a**) Hydrogen peroxide staining of JIMT-1 cells after Se treatments; (**b**) hydrogen peroxide staining of BT-474 cells after Se treatments. After acclimatization for 24 h, 50 units/well of SOD and 100 units/well of catalase were added to all wells in media. All cells, control, selenite, Tz and Se-Tz were appropriately treated and then DCFH-DA was added to the cells at a final concentration of 30 μM in media. Fluorescence intensity was read using a fluorescence microplate reader with an excitation at 475 nm and emission at 525 nm. Selenite was used as a redox toxic positive control at a concentration of 10 µgSe/well. Selenite at concentrations less than 10 µgSe/well had no visible effect on cancer cell lines under these experimental conditions.

Following in a manner similar to the one described above for the DHE detection of O_2_^•−^, JIMT-1 and BT-474 cells were subjected to dichlorofluorescein (DCFH-DA) staining and treatment with Tz, selenite (10 μgSe/well) and Se-Tz (4.8 μgSe) for the detection of hydrogen peroxide (H_2_O_2_). Protein concentrations for the Se-Tz and Tz treatments were the same. The results of the photographic detection of the green fluorescence from the deacetylation of DCFH-DA by a cellular esterase to a nonfluorescent compound is followed by the intracellular ROS oxidation of DCFH-DA to 2′,7′-dichlorofluorescein (DCF), which is highly fluorescent as shown in Figure 6. Assuming that the H_2_O_2_ generated green fluorescence observed in Figure 6 from DCFH is derived from the selenium treatments initially generating O_2_^•−^ as shown in Figure 5, cellular SOD then generates the H_2_O_2_ from O_2_^•−^ with the differences in fluorescence between the two cell lines being striking. JIMT-1 control and Tz treated cells reveal almost no H_2_O_2_ being generated, while selenite and Se-Tz treated cells result in large amounts of H_2_O_2_ being generated as measured by DCFH-DA (Figure 6a). As suggested from the DHE experiments in Figure 5, JIMT-1 cells are perhaps resistant to Tz, as Tz produces little O_2_^•−^ over control cells and any cellular H_2_O_2_ is rapidly converted to water by either catalase or glutathione peroxidase or both, eliminating Tz toxicity not seen with selenite or Se-Tz treatments (Figure 6a). Unlike the JIMT-1 Herceptin^®^ resistant control cells, the BT-474 Herceptin^®^ sensitive control cells show a greater background H_2_O_2_ generation, which is visually enhanced by selenite, Tz and Se-Tz (Figure 6b). The visual enhancement of H_2_O_2_ by Tz may account for the reported BT-474 cell Herceptin^®^ susceptibility to Tz. Both selenite and Se-Tz visually enhance H_2_O_2_ generation even more and visually change the morphological “island” pattern of the BT-474 control cells, seen in Figure 1 and Figure 6b, which are “lit up” by the background H_2_O_2_ produced by the control BT-474 cells. Following photography, fluorescence by DCF activation at 475 and 525 nm was performed with the microplate reader. The DCF florescence intensity in all but the JIMT-1 control and Tz treated cells was too intense to be quantified by the plate reader and for that reason no quantitative florescence data are reported. 

### 2.6. Apoptotic Caspase-3 Activity Assay

**Figure 7:**
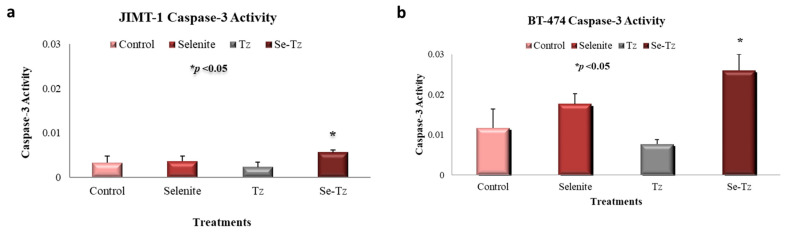
Caspase-3 activity from cell lines after selenium treatments. (**a**) JIMT-1 cells after 24 h of Trastuzumab and selenium treatments; (**b**) BT-474 cells after 72 h of Trastuzumab and selenium treatments. Cells were treated with Se-Tz at the concentration of 4.8 µgSe/well and Tz cells were treated with an equal mab protein concentration. Selenite was used as a redox toxic positive control at a concentration of 10 µgSe/well. Selenite at concentrations less than 10 µgSe/well had no visible effect on cancer cell lines under these experimental conditions.

JIMT-1 cells were seeded at the density of 3 × 10^5^ cells/well in 24-well plates and then treated with native Tz, Se-Tz at 4.8 µgSe/well and selenite, 10 µgSe/well for 24 h. Se-Tz treated cells demonstrated a significant (* *p* < 0.05) 2-fold increase in caspase-3 activation as compared to native Tz (Figure 7a). There was no significant increase in caspase-3 in the selenite treated cells compared to control and Tz treated cells. BT-474 cells were seeded at a density of 3 × 10^5^ cells/well in 24-well plates and treated with native Tz, Se-Tz at 4.8 µgSe/well and selenite, 10 µgSe/well for 72 h. For BT-474 cells, as indicated by caspase-3 units in Figure 7b, Se-Tz significantly increased caspase-3 (* *p* < 0.05) activation by nearly 3-fold over control cells after 72 h of incubation. Selenite treated cells increased caspase-3 activation by 2-fold as compared to control and native Tz treated cells, but the increase was not significant—*p* < 0.05.

### 2.7. Cell Viability with the MTT Assay 

**Figure 8:**
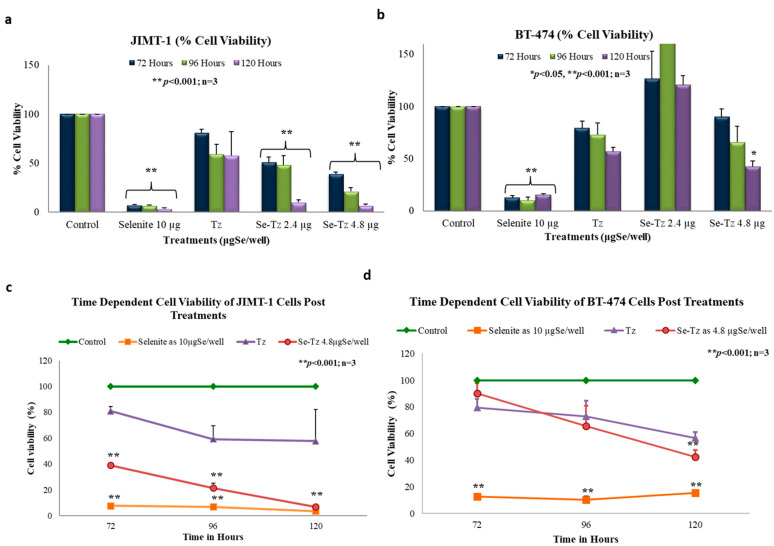
Cells were treated with Se-Tz at the concentrations of 2.4 and 4.8 µgSe/well and Tz cells were treated with an equal mab protein concentration. Selenite was used as a redox toxic positive control at a concentration of 10 µgSe/well with significant decrease in % cell viability. MTT cell viability assay demonstrated significant decrease in % cell viability in Se-Tz treated JIMT-1 cells (**a**) and BT-474 cells (**b**) in a time and dose dependent manner compared to control cells. Se-Tz at 2.4 µgSe/well concentration up-regulated % cell viability of BT-474 cells (**b**). Figure 8 (**c**,**d**) represent the time dependent decrease in % cell viability of JIMT-1 and BT-474 cells, respectively, of Se-Tz treated cells at the concentration of 4.8 µgSe/well and selenite at 10 µgSe/well compared to control cells. Tz cells were treated with an equal mab protein concentration. Selenite at concentrations less than 10 µgSe/well had no visible effect on both the cancer cell lines under these experimental conditions.

**Figure 9:**
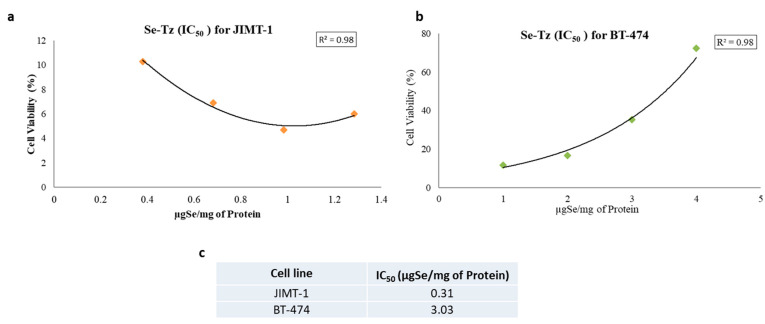
(**a**) IC_50_ calculations for Se-Trastuzumab treated JIMT-1 cells from Figure 8d and (**b**) IC_50_ calculations for Se-Trastuzumab treated BT-474 cells from Figure 8e. (**c**) The IC_50_ is calculated here for Se-Tz after treating the two cancer cell lines from low to high concentrations using the Graph Pad Prism software from 9a and 9b.

The MTT assay was used as a supportive measure of cell viability of control cells and Se toxicity as assessed by Trypan Blue exclusion photographically in Figure 4. Both cell lines, the Tz Herceptin^®^ resistant JIMT-1 and Tz Herceptin^®^ sensitive BT-474 cells, were seeded at a density of 1 × 10^5^ cells and treated with two different concentrations of Se-Tz; 2.4 and 4.8 µgSe/well; as well as Tz and selenite, 10 µgSe/well. To ascertain the effect of time, cells were incubated post-treatments for 72, 96 and 120 h (Figure 8a,b,d). The cytotoxic effect of Se-Tz on cell viability was demonstrable in a dose and time dependent manner in both cell lines. At the highest concentration tested, Se-Tz at 4.8 µgSe/well induced a significant decrease in the proliferation rate of the Tz resistant JIMT-1 cell line (** *p* < 0.001) as compared to the Tz treated and control cells (Figure 8a). It was also interesting to observe the time dependent effect on the cell proliferation at 120 h, for upon treatment Se-Tz with 2.4 µgSe/well and 4.8 µgSe/well, loss of cell viability was nearly equivalent. Tz treated JIMT-1 cells did not show any significant (*p* > 0.05) reduction in cell viability in accordance with the known Tz resistance of the JIMT-1 cells [18] Selenite at 10 µgSe/well revealed cell viability to be less than 35% after 72, 96 and 120 h of incubation. JIMT-1 cells were also found to be more susceptible to selenite toxicity as compared to the Tz sensitive BT-474 cells. The Tz sensitive BT-474 cells demonstrated reduction in cell viability only at the highest concentration of Se-Tz tested—4.8 µgSe/well—in a time dependent manner, as shown in Figure 8b. An actual increase in the rate of cellular proliferation at lower concentrations of Se-Tz, less than 2.4 µgSe/well, was observed, demonstrating some Se resistance. Selenite at 10 µgSe /well significantly (*p* > 0.001) reduced cell viability of both cell lines to less than 10% to that of control cells. The susceptibility of treatments of the two cell lines by Se-Tz were different (Figure 9). This was seen in the calculation of both cell lines, JIMT-1 (0.310) and BT-474 (3.03). Graph pad prism software was used to calculate the IC_50_ values of Se-Tz treatments of the two cell lines. Graphical depiction of the IC_50_ was performed using the Microsoft Excel, 2007, software. 

### 2.8. Scanning Electron Micrographs of JIMT-1 and BT-474 Cells

**Figure 10:**
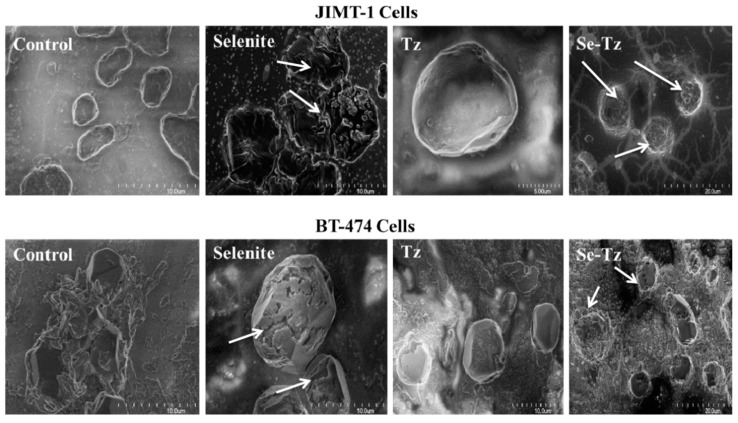
Morphology of control and selenium treated JIMT-1 and BT-474 cells by scanning electron microscopy. Representative control and Se treated cancer cells at 72 h were fixed in 10% buffered formalin on glass cover slips and allowed to air-dry for 4–5 h. SEM images of both the cancer cell lines were taken using a Hitachi Model S-4700 FE-SEM at magnifications of 5–20 µm after 96 h of selenium treatment.

JIMT-1 and BT-474 cells seeded at the density of 1 × 10^5^ cells/well in 24-well plates and treated with Tz, Se-Tz at 9.6 µgSe/well and selenite at 10 µgSe/well for 96 h. Selenium treated and untreated cells were collected from the wells by pipette and were fixed in 10% buffered formalin on glass coverslips followed by 4–5 h of air-drying. SEM images were taken at 5–20 µm and photos were stored on a Hitachi computer (Figure 10). Se-Tz and selenite treated cells demonstrated disrupted membranes in both cell lines as compared to Tz or control cells, which revealed smooth cellular membranes. Selenite treatment also demonstrated intense membrane disruption with loss of original cellular morphology as compared to control cells. More apparent cytotoxic effects of Se-Tz and selenite to cell morphology were visible in both JIMT-1 and BT474 cell lines, consistent with free radical generation of O_2_^•−^ and H_2_O_2_ previously demonstrated and now visualized in the representative SEM micrographs (Figure 10).

## 3. Discussion

Breast cancer remains the most common invasive cancer and cause of death among women, with 279,100 estimated new cases reported in 2020 and ~42,690 deaths (ACS, Surveillance Data of Breast Cancer, 2019). Approximately 20–30% of these breast cancers are Her/2+ [19] and, before the 1990s, women had poor survival rates as surgery was and remains a highly employed modality of prevention (i.e., BRACA) and treatment of breast cancer. With the discovery of the EGFR1 receptor, the Her/2 gene and the clinical development of monoclonal antibodies, in September 1998, the FDA approved the first clinical “neutralizing” mab to treat Her/2+ breast cancer, Trastuzumab (Tz), better known publically as Herceptin^®^. Many women responding to Herceptin^®^ treatment, a characteristic of the BT-474 cells used in this study, only have their cancer become resistant with disease progression [19,20], more characteristic of the JIMT-1 cells used in this study. In response to clinical Tz resistance, Genentech-modified Tz by the covalent addition of three or more molecules of the chemotherapeutic cytoskeletal inhibitor, emtansine. This first clinical Antibody–Drug Conjugate (ADC) T-DM-1, Kadcyla^®^, significantly extended the survival of women with Her/2+ Tz resistant advanced breast cancer [20]. T-DM-1 was approved by the FDA in 2013 as the successor to the clinical use of Tz against Her/2+ breast cancer and patients receiving T-DM-1 treatment have shown disease free progression with improved survival rates [21,22]. Despite the treatment responses with T-DM-1, most patients eventually develop progressive disease with few patients showing long term stability with Her/2+ directed monoclonal breast cancer antibody therapy. 

In this research we have sought to improve upon, compare and demonstrate improvement in cancer cell inhibition with Se-Tz treatments, compared to Tz treatments in vitro, against the Herceptin^®^ susceptible BT-474 cells and the Herceptin^®^ resistant JIMT-1 cells. This observational improvement is clearly demonstratable by Trypan Blue light microscopy of cell morphology, MTT and caspase-3 assays and SEM photographs. The difference between Tz and Se-Tz cancer cell growth inhibition is attributable to the covalent addition of selenium forming the colored Se-Tz derivative of Herceptin^®^ shown in Figure 2. The covalent Se attachment transforms Herceptin^®^ and other mabs, from being “neutralizing” mabs against Her/2+ cancer cells into a “cytotoxic” Se-ADC, catalytically generating superoxide, O_2_^•−^.

The Se-Tz generated O_2_^•−^ is demonstrable in vitro by a Lucigenin Chemiluminescent Assay and ex vivo intracellularly by the florescent dye Dihydroethidium. H_2_O_2_ was also detectable ex vivo following selenium treatment of cell lines with Dichlorodiflurohydrazine when H_2_O_2_ was detected by DCFH-DA it was likely due to the intracellular reduction in the selenium generated O_2_^•−^ by Se-Tz. 

This research was conducted in a fashion similar to Goswami et al. [23] who reported on Se-Transferrin against leukemia cell lines and Khandelwal et al. for their comparison of Se-Herceptin^®^, and Avastin^®^ against Triple Negative Breast Cancer (TNBC) cell lines [24]. The experimental results observed herein and conclusions we draw from the present data are very similar to the conclusions drawn from the above referenced publications, [23] and [24] and they are:

(1) Redox selenium covalently attached to any mab generates O2•−., conferring the native mab, or protein, cytotoxic, as is selenite generating O2•−, used concurrently in experiments as a toxic positive selenium control;
(2) Se-Tz was more toxic to both cell lines, as was selenite, than Tz in a dose and time dependent manner across all measurements of cell morphology and metabolic viability;
(3) Se-Tz was ~10X more cytotoxic (IC50) (Figure 9) to the Tz resistant JIMT-1 cells than the Tz susceptible BT-474 cells, (JIMT-1; 0.310 µgSe) and BT-474 (3.03 µgSe) (Figure 9 c),
(4) Cell death from redox selenium being time and dose dependent is likely induced apoptosis from the oxidative stress of O2•−, being intracellularly generated by the oxidation of mitochondrial and cytoplasmic thiols;
(5) The collective data for Se-Tz treatments of the JIMT-1 and BT-474 cancer cells is fully consistent with the SEM cell data of Spallholz et al. [25] showing that only redox cycling selenium, superoxide (O2•−) generating selenium compounds are cytotoxic to mammalian, bacteria and yeast cells [26,27].

In conclusion, this research has demonstrated Se-Tz synthesized from a coupling reaction from a Bolton–Hunter-like selenoester with Tz significantly decreased cell viability in Her/2+ JIMT-1 and BT-474 cell lines in a dose and time dependent manner (** *p* < 0.001, * *p* < 0.05). Trypan blue staining and an MTT assay at various Se-Tz concentrations demonstrated significant, * *p* < 0.05 loss of JIMT-1 and BT-474 cell viability. The Caspase-3 assay demonstrated that reduced cell viability of both Her/2+ cell lines was due to either extrinsic (death ligand) or intrinsic (mitochondrial) apoptotic pathways in both JIMT-1 and BT-474 cell lines (* *p* < 0.05). Since Se redox chemistry is known to cause mitochondrial swelling [28] apoptosis is likely induced by redox selenium intrinsically. Moreover, the DHE assay demonstrated Se-Tz induced intracellular superoxide generation in JIMT-1 and BT-474 cells in a dose dependent manner (* *p* < 0.05). These results suggest the possibility of a drug delivery system of redox Se by Trastuzumab to Her/2+ breast cancer patients before or after patients encounter resistance. In addition, conjugating redox selenium selenides to Tz, and other proteins suggest a much wider application of this redox technology to other antibodies and targeting molecules. In the contemporaneous situation of COVID-19 the technology is suggestive that a SARS-CoV-2 mab with redox selenium could be employed with a more toxic viral capacity than with an S-protein or other neutralizing SAR-2 mabs alone.

Herceptin^®^ is a registered trademark of Genentech (Roche) for Tz antibody. 

## 4. Materials and Methods

### 4.1. Reagents

The *N*-hydroxysuccinimide ester of 3-selenocyanopropionic acid (a modified selenium Bolton–Hunter Reagent; BHR) was synthesized by Eburon Organics N. V., Belgium following a previously reported method [23,29] by a *N,N’*-dicyclohexylcarbodimide (DCC) assisted coupling reaction of *N*-hydroxysuccinimide and 3-selenocyanopropionic acid. The final Se-BHR ester product has a brick red color (Figure 2a). Sodium selenite was from Sigma-Aldrich Chemical Company, St. Louis, MO, USA. All other chemicals were reagent grade or purchased as chemical kits from their respective manufacturers.

### 4.2. Selenium Antibody Conjugation

Selenium Analysis of Se-Tz and Tz followed by an In Vitro Chemiluminescent Assay for Superoxide. 

Following Tz conjugation with redox selenium and dialysis, the pH was verified to be pH 7.4, and aliquots of Se-Tz and Tz sent to TraceAnalysis Inc. were determined by ICP-MS to contain 25.8 µgSe/mg protein and <0.35 µgSe/mg protein, respectively. Both Se-Tz and Tz were assessed for their ability to generate (O_2_^•−^) using an in vitro Lucigenin Chemiluminescent assay (Figure 3) at 37 °C degrees as previous described by Chen et al. [16]. 

### 4.3. Cell Culture of Breast Cancer Cells 

JIMT-1 cells were purchased from Addexbio (Cat #C0006005, San Diego, CA, USA) propagated and maintained in DMEM-F12 (ATCC Cat# 11320-033, Manassas, VA, USA) with fetal bovine serum added to a final concentration of 10%. BT-474 cells were purchased from ATCC (Cat # HTB-20, Manassas, VA, USA) propagated and maintained in RPMI 1640 media (ATCC Cat# 30-2001, Manassas, VA, USA) with fetal bovine serum added to a final concentration of 10%. Both cell lines (Figure 1) were grown in tissue culture flasks in a humidified atmosphere of 95% air and 5% CO_2_ at 37 °C. Growth medium was renewed every 2 or 3 days and cell densities were maintained at 1 × 10^5^ cells/mL before passage. Under these incubation conditions it had been determined that after seeding of equal amounts of cells, the rates of cell division of the JIMT-1 cells were ~ 2X that of the BT-474 cells.

### 4.4. Trypan Blue Viable Cell Assay

Both cell lines were seeded in triplicates as described above at a concentration of 1 × 10^5^ cells/well in 24-well plates. Upon the completion of the control and selenium concentration addition and treatment times as indicated in Figure 4a,b, photographs of the cells were taken at 20X magnification using an EVOS microscope (AMEX1000, Life Technologies, Grand Island, NY, USA) following addition of Trypan Blue, 10 µL/well of the TB stock solution. 

### 4.5. Dihydroethidium (DHE), Dichlorofluorescein (DCFH-DA) and Caspase-3 Assays

JIMT-1 cells were seeded in DMEM-F12 (Thermo Fisher Scientific Cat # 21041-025) in phenol red free medium and BT-474 cells were seeded in RPMI 1640 (Sigma-Aldrich Cat # R7509) phenol red free medium. Both cell lines were grown in triplicates to a density of 1 × 10^5^ cells/well in 24-well plates. For the DHE, DCFH-DA and caspase-3 assays, JIMT-1 cells were seeded at a concentration of 2 × 10^5^ cells/well and the BT-474 cells were seeded at a concentration 3 × 10^5^ cells/well. The concentration of cells was different having determined, as noted above, that JIMT-1 cells divide almost twice as rapidly as BT-474 cells. In triplicates, cells were treated with Se-Tz at Se concentrations between 0.6 µg and 19.2 µgSe/well for 24, 72, 96, and 120 h. All experimental data with Se-Tz was compared to data from control cells, Tz treated cells, and cells treated with sodium selenite (10 µgSe/well).

### 4.6. Superoxide Detection Assay: Dihydroethidium (DHE)

JIMT-1 and BT-474 cells were seeded in triplicates at a density of 2 × 10^5^ cells/well in 24-well plates. After acclimatization for 24 h, 50 units/well of Superoxide dismutase (SOD) (Sigma-Aldrich Cat# S7571, St. Louis, MO, USA) from bovine erythrocytes and 100 units/well of catalase from bovine liver (Sigma-Aldrich Cat# C1345-1G, St. Louis, MO, USA) were added to all wells in media. All cells, control, Se-Tz, Tz, selenite along with a treatment blank, received DHE in media at a final concentration of 10 µM/well. Cells were photographed at 4X and 20X 30 min after treatment with an EVOS microscope for the red fluorescence generated by O_2_^•−^. Visual photography was immediately followed by measurement of fluorescence intensity using a fluorescence microplate reader (BioTekSynergy H1, Winooski, VT, USA) with an excitation at 520 nm and emission at 610 nm (Figure 5).

### 4.7. Hydrogen Peroxide Detection Assay: Dichlorodihydrofluorescein Diacetate (DCFH-DA) Fluorescent Assay 

JIMT-1 and BT-474 cells were seeded in triplicates at a density of 2 × 10^5^ cells/well in 24-well plates. After acclimatization for 24 h, 50 units/well of superoxide dismutase (SOD) (Sigma-Aldrich Cat# S7571, St. Louis, MO, USA) from bovine erythrocytes and 100 units/well of catalase from bovine liver (Sigma-Aldrich Cat# C1345-1G, St. Louis, MO, USA) were added to all wells in media. All cells, control, Se-Tz, Tz, selenite along with a treatment blank, received DCFH-DA in media (Sigma-Aldrich Cat#4091-99-0, St. Louis, MO, USA) at a final concentration of 30 µM/well. Cells were incubated with the dye for 15 min followed by media removal from each well. The cells were then photographed at 4X after 15–20 min of treatment incubation with an EVOS microscope for the green fluorescence generated by H_2_O_2._ Fluorescence intensity was read using a fluorescence microplate reader (BioTekSynergy H1, Winooski, VT, USA) with an excitation at 475 nm and emission at 525 nm (Figure 6).

### 4.8. Apoptotic Assays: Caspase-3 

JIMT-1 and BT-474 cell lines were seeded in triplicate at a density of 3 × 10^5^ cells/well in 24-well plates and incubated with Se-Tz, Tz and selenite treated cells for 24 and 72 h, respectively. Control and treated cells were lysed with buffer (Abcam Cat#39401 Cambridge, MA, USA) and protein concentrations were measured using the BCA kit (Pierce^TM^ #23225 Waltham, MA, USA). Cells were measured for caspase-3 by p-NA light emission at 405 nm using a microtiter plate (BioTek Synergy H1, Winooski, VT, USA) (Figure 7).

### 4.9. MTT Cell Viability Assay

Cells were seeded in triplicate in 24-well plates at a density of 1 × 10^5^ cells/well and 3-[4, 5-dimethylthioazol-2-yl]-2-5-diphenyltetrazolium bromide (MTT) solution (5 mg/mL) 10% (*v*/*v*) was added after treatment to each well (Figure 8a,b). The formazan generated by viable cells was solubilized by Tween-20 and isopropanol (1 mL/well) and absorbances of the soluble formazan at 570 and 690 nm were recorded using a microplate reader (BioTek Synergy H1, Winooski, VT, USA). 

### 4.10. Se-Tz IC_50_ Data

The IC_50_ for Se-Tz treated JIMT-1 and BT-474 cells was determined by using the cell viability data in dose response equations built into the Graph Pad Prism software and the graphs were plotted using Microsoft Excel (Figure 9). 

### 4.11. Scanning Electron Microscopy: Imaging

JIMT-1 and BT-474 cells were seeded at the density of 1 × 10^5^ cells/well in 24-well plates followed by the treatments with Se-Tz at 9.6 µgSe/well, Tz or selenite, 10 μgSe/well. At 96 h post treatment, 20 µL of cells was removed with an Eppendorf pipette and was fixed in PBS buffered (10%) formalin (pH 7.4) on 2 × 2 cm glass cover slips (Fisher brand Cat#12-545-A, Pittsburg, PA, USA). The cells were allowed to air-dry for 4–5 h and images of cells were taken at 5–20 µm of magnification and saved on a computer from Hitachi, Model S-4700 FE-SEM (Wallingford, CT, USA) (Figure 10).

### 4.12. Statistical Analyses

All experimental assays were conducted in triplicate or more replications unless otherwise noted and are representative of three or more independent experiments. Statistical results are expressed as the Mean ± one Standard Deviation (SD). Analyses were performed using the SPSS statistical software with two-tailed paired t-tests, an independent t-test and one-way ANOVA. Significance between and among treatments is indicated in the text and Figures as *p* < 0.05 (*) and *p* < 0.001 (**).

## Data Availability

Not applicable.

## Abbreviations

BHR	Bolton–Hunter Reagent
CL	Chemiluminescence
DCC	*N,N’*-Dicyclohexylcarbodimide
DCFH-DA	Dichlorodihydrofluorescein Diacetate
DHE	Dihydroethidium
ECL	Enhanced Chemiluminescence
EGFR	Epidermal Growth Factor Receptor
FDA	Food and Drug Administration
GSH	Glutathione
Her/2	Human Epidermal Growth Factor Receptor 2
H_2_O_2_	Hydrogen Peroxide
IC50	50% Inhibitory Concentration
mab(s)	Monoclonal Antibody(ies)
MTT	Tetrazolium dye 3-(4,5-dimethylthiazol-2-yl)-2,5-diphenyltetrazolium bromide
O_2_^•−^	Superoxide
ROS	Reactive Oxygen Species
Se	Selenium
SEM	Scanning Electron Microscope
Se-Tz	Seleno-Trastuzumab
SOD	Superoxide Dismutase
TB	Trypan Blue
THF	Tetrahydrofuran
Tz	Trastuzumab, Herceptin^®^
T-DM-1	Kadcyla^®^
